# Addressing the Impact of Noncommunicable Diseases and Injuries (NCDIs) in Ethiopia: Findings and Recommendations from the Ethiopia NCDI Commission

**DOI:** 10.4314/ejhs.v32i1.18

**Published:** 2022-01

**Authors:** Solomon Tessema Memirie, Wubaye Walelgne Dagnaw, Mahlet Kifle Habtemariam, Alemayehu Bekele, Dejuma Yadeta, Amsalu Bekele, Wondu Bekele, Molla Gedefaw, Mathewos Assefa, Mieraf Taddese Tolla, Awoke Misganaw, Neil Gupta, Gene Bukhman, Ole F Norheim

**Affiliations:** 1 Department of Pediatrics and Child Health, College of Health Sciences, Addis Ababa University, Addis Ababa, Ethiopia; 2 Department of Global Public Health and Primary Care, University of Bergen, Bergen, Norway; 3 Federal Ministry of Health of Ethiopia, Addis Ababa, Ethiopia; 4 Ethiopian Public Health Association, Addis Ababa, Ethiopia; 5 Department of Internal Medicine, College of Health Sciences, Addis Ababa University, Addis Ababa, Ethiopia; 6 Mathiwos Wondu-YeEthiopia Cancer Society, Addis Ababa, Ethiopia; 7 Departement of Oncology, College of Health Sciences, Addis Ababa University, Addis Ababa, Ethiopia; 8 Department of Global Health and Population, Harvard T.H. Chan School of Public Health, Boston, MA, USA; 9 Ethiopia Public Health Institute, Addis Ababa, Ethiopia; 10 Program in Global NCDs and Social Change, Department of Global Health and Social Medicine, Harvard Medical School, Boston, MA, USA; 11 NCD Synergies, Partners in Health, Boston, MA, USA

**Keywords:** Noncommunicable diseases and injuries, priority-setting, NCDI interventions, low-income countries, Global and National NCDI commission, Ethiopia

## Abstract

**Background:**

Noncommunicable diseases and injuries (NCDIs) are the leading causes of premature mortality globally. Ethiopia is experiencing a rapid increase in NCDI burden. The Ethiopia NCDI Commission aimed to determine the burden of NCDIs, prioritize health sector interventions for NCDIs and estimate the cost and available fiscal-space for NCDI interventions.

**Methods:**

We retrieved data on NCDI disease burden and concomitant risk factors from the Global Burden of Disease (GBD) Study, complemented by systematic review of published literature from Ethiopia. Cost-effective interventions were identified through a structured priority-setting process and costed using the One Health tool. We conducted fiscal-space analysis to identify an affordable package of NCDI services in Ethiopia.

**Results:**

We find that there is a large and diverse NCDI disease burden and their risk factors such as hypertension and diabetes (these conditions are NCDIs themselves and could be risk factors to other NCDIs), including less common but more severe NCDIs such as rheumatic heart disease and cancers in women. Mental, neurological, chronic respiratory and surgical conditions also contribute to a substantial proportion of NCDI disease burden in Ethiopia. Among an initial list of 235 interventions, the commission recommended 90 top-priority NCDI interventions (including essential surgery) for implementation. The additional annual cost for scaling up of these interventions was estimated at US$550m (about US$4.7 per capita).

**Conclusions:**

A targeted investment in cost-effective interventions could result in substantial reduction in premature mortality and may be within the projected fiscal space of Ethiopia. Innovative financing mechanisms, multi-sectoral governance, regional implementation, and an integrated service delivery approach mainly using primary health care are required to achieve these goals.

## Introduction

Noncommunicable diseases (NCDs) are the leading cause of premature mortality in lower- and middle-income countries (LMICs), accounting for 60% of deaths (1). Injuries are furthermore the cause of an additional 9% of deaths (1). NCDs have become important and growing causes of health inequality and inequity (2,3). Poorer and less-educated people are disproportionately affected by NCDs (2). NCDs, as a result of the high out-of-pocket expenditure on health and long-term care, impose a major economic burden on households, and the catastrophic level of medical expenses associated with NCDs are more likely to be experienced by the poor than by the rich (4).

Noncommunicable diseases and injuries (NCDIs) have become a major public-health problem in Ethiopia, resulting in 44% of total annual mortality (1). Ethiopia has experienced one of the most rapid shifts in NCD burden globally with an estimated 65% of disability-adjusted life years (DALYs) attributable to NCDs by 2040 and is among the countries least prepared for this transition (5). Despite rapid economic gains in the past decade, Ethiopia is one of the world's poorest countries, with a GNI per capita of US$740 in 2017 and 26.7% of the population living on less than US$1.90 per day (6). Per capita health expenditure was US$33 in 2016/2017 — far less than the US$112 per capita suggested to attain the health-related Sustainable Development Goals (SDGs) in low-income countries (7,8).

SDG 3.4 requires countries to commit to reducing premature mortality from NCDs by a third, through prevention and treatment (9).

Despite commitment pledged by countries in the 2011 UN political declaration on the Prevention and Control of NCDs (UN high-level meeting), progress to combat NCDs has so far been slow, particularly in regards to populations living in extreme poverty (10,11). To catalyse the commitments with regard to NCDs, the Lancet Commission on Reframing Noncommunicable Diseases and Injuries (NCDIs) for the Poorest Billion was launched in 2015 (12). It aims to achieve a better understanding of how NCDIs affect those living in extreme poverty and how to address the problem in low-income settings, and to provide recommendations that can be applied to both international and national settings. With the support of the Lancet NCDI Poverty Commission and the University of Bergen, the Ethiopia NCDI Commission was established in August 2016 to utilize existing data and a structured priority setting process by a panel of local experts to identify priority NCDI conditions in Ethiopia, select priority evidence-based interventions for introduction and scale, determine the potential costs of these interventions, and project the fiscal space available to finance such interventions under various scenarios. This Commission published a full set of findings and recommendations in a national dissemination meeting in November 2018 (13). Here, we present a summary of this Commission's methodology, key findings and recommendations.

## Materials and Methods

***NCDI disease burden:*** We retrieved data on NCDI disease burden and the concomitant risk factors from the Global Burden of Disease (GBD) Study, complemented by a systematic review of published literature from Ethiopia. We searched the following databases using a mix of search terms: Global Burden of Disease/IHME, PubMed, Cochrane, HINARI, Google Scholar, EMBASE, The World Bank, WHO Regional Databases and local journals, including grey literature. We included articles published from 1990 to December 2017 that had clear objectives and methodologies and addressed the major NCDIs and their risk factors. We summarised the evidence for each NCD, injury and risk factor. Where several prevalence studies of similar populations were found, we used a weighted average. In this review, community- and institution-based studies and some hospital-based studies were used to show population prevalence, whilst mortality studies and hospitalisation studies were used to indicate disease severity and outcomes.

**Priority setting process**: Following identification of the major NCDIs in Ethiopia, interventions were selected and prioritised in a four-step process. Firstly, priority-relevant evidence from national and international studies was collected, critically reviewed, and supplemented with expert judgements. Secondly, this evidence, along with the WHO principles, was used to prioritise interventions, ranking them in three categories (top, medium and low priority interventions) (14). The WHO principles for prioritising interventions were maximisation of health in the total population (refers to the most cost-effective (CE) interventions), priority to the worse-off and financial risk protection (FRP) (14). Thirdly, the interventions categorised as being top-priority were costed using the OneHealth Tool (15). Fourth, based on cost estimates, fiscal space analysis and budget scenarios, the Commission revised the list to fit within the budget, using the WHO principles. Based on the CE of the intervention, with extra priority to the worse off and high FRP; the commission developed the following three categories to prioritise the NCDI interventions:

1. Top priority NCDI interventions: Interventions with CE ratio < 0.5 x GDP per capita, weighed for priority to the worse off in health and FRP
2. Medium priority NCDI interventions: Interventions with CE ratio 0.5–1 x GDP per capita, weighed for priority to the worse off in health and FRP
3. Low priority NCDI interventions: Interventions with CE ratio > 1 x GDP per capita), not targeting the worse off in health and low FRP.

**Estimation of costs and fiscal space**: Cost by intervention was estimated using OneHealth Tool version 4.5 using the software's default data on cost of drugs and supplies and the default population model for Ethiopia (15). Unit costs presented in a literature review published as appendices to DCP3 publications were used when cost data was not available in OneHealth Tool. Program costs (such as training, supervision, and construction of new facilities) are not included in our first cost estimates. We therefore added 10% to the total cost estimated to account for programme costs (mostly for training and supervision). In order to assess local capacity with regard to generating resources for health, the Commission performed a simple fiscal-space analysis. Through definition of reasonable assumptions and targets, fiscal-space projections were developed under three different scenarios: base case, medium and best case. Details of the methodology are presented in the commission's full report (13).

**Estimated health impact of NCDI interventions**: To estimate impact, we built on the DCP3 methods and framework developed by Watkins, *et al.* (16). We estimated the impact of implementing the Highest Priority Package (HPP) for NCDI services (Table 3). These packages are described in Jamison *et al.* (17). We assumed that these packages are implemented to 80% coverage by 2030, and we assume high quality of service provision (= 80% efficiency). There is therefore some risk of overestimation. For further details, see Watkins *et al* (16).

**Table 3:** Top-priority NCDI interventions, listed by disease area and delivery platform, 2019–2023

Disease Area	Interventions	Delivery Platform
**Cancer**	HPV vaccine to prevent cervical and anal cancer	Community
**Cancer**	Hep B vaccine to prevent liver cancer	Health centre
**Cancer**	Visual inspection with acetic acid (VIA) and cryotherapy for precancerous lesions	Health centre
**Cancer**	Diagnosis without screening for breast cancer	District hospital
**Cancer**	Breast cancer treatment: Stage I	Referral/specialist hospital
**Cancer**	Cervical cancer treatment: Stage I	Referral/specialist hospital
**Cancer**	Emergency surgery for obstruction, colon cancer	District hospital
**Cancer**	Treat selected cancers in paediatric cancer units/hospitals (Leukaemia, retinoblastoma)	Referral/specialist hospital
**Cancer**	Basic palliative care, breast cancer	Community
**Cancer**	Basic palliative care, cervical cancer	Community
**Cancer**	Basic palliative care, colon cancer	Community
**CVD & Resp.**	Encourage adherence to medications	Community
**CVD & Resp.**	Community-based opportunistic screening for CVD	Community
**CVD & Resp.**	Primary prevention for those with absolute risk of CVD>10%	Health centre
**CVD & Resp.**	Treatment of cases with established ischaemic heart disease (secondary prevention)	Health centre
**CVD & Resp.**	Treatment of cases with established cerebrovascular disease (secondary prevention)	Health centre
**CVD & Resp.**	Treatment of cases with acute pharyngitis to prevent rheumatic fever	Health centre
**CVD & Resp.**	Treatment of cases of rheumatic heart disease (with benzathine penicillin)	Health centre
**CVD & Resp.**	Management of diabetes mellitus type 2	Health centre
**CVD & Resp.**	Revascularisation or amputation for limb ischaemia	District hospital
**CVD & Resp.**	Management of acute heart failure with diuretics and noninvasive positive-pressure ventilation	District hospital
**CVD & Resp.**	Insulin management of diabetes mellitus type 1	District hospital
**CVD & Resp.**	Asthma: Low-dose inhaled beclometasone + SABA	District hospital
**CVD & Resp.**	COPD: Exacerbation treatment with antibiotics	District hospital
**CVD & Resp.**	Cardiac surgery for rheumatic heart disease Referral/specialised hospital
**MNS**	Dietary supplement of folic acid and iron for pregnant women	Community
**MNS**	Identification of children with MNS disorders in schools	Community
**MNS**	Safer storage of pesticides in the community/households	Community
**MNS**	Home visits to reduce the risk of postpartum depression	Community
**MNS**	Psychosocial care for perinatal depression	Health centre
**MNS**	Depression: Basic psychosocial care and antidepressant medication for first episode of moderate to severe cases and episodic and/or maintenance treatment for recurrent cases	Health centre
**MNS**	Anxiety: Basic psychosocial treatment and antidepressant medication for anxiety disorders (moderate to severe cases)	Health centre
**MNS**	Psychosis: Psychosocial support and antipsychotic medication	Health centre
**MNS**	Bipolar disorder: Psychosocial support, advice, and followup for bipolar disorder, plus mood-stabilising medication	Health centre
**MNS**	Pesticide intoxication management	Health centre
**MNS**	Epilepsy: Follow-up and anti-epileptic medication	Health centre
**MNS**	Alcohol use disorders: Diagnosis, management of withdrawal, relapse prevention with medication	District hospital
**MNS**	Management of opioid withdrawal	District hospital
**MNS**	Electroconvulsive therapy for severe or refractory depression	Referral/specialist hospital
**Surgical**	Pre-hospital care of injuries	Community
**Surgical**	Management of injuries	District hospital
**Surgical**	Management of general emergency surgical conditions	District hospital
**Surgical**	Management of obstetric and gynaecological surgical emergencies	District hospital
**Surgical**	Management of congenital surgical problems	Referral/specialist hospital
**Surgical**	Cataract surgery	District hospital

## Results

**NCDI disease burden and the concomitant risk factors in Ethiopia**: Data on NCDI disease burden and the concomitant risk factors was retrieved from the Global Burden of Disease (GBD) Study, complemented by a systematic review of published literature from Ethiopia. The GBD data showed that in 2017 44.4% of total mortality in Ethiopia was due to NCDIs (1). Cardiovascular diseases and cancer constitute an estimated 43% of NCDs and injury mortality. Among individuals between the ages of 50–69 years, NCDI is a cause for 70% of the mortality. The same GBD study showed that NCDIs caused a substantial proportion of the total Disability-Adjusted Life Years (DALYs) lost (40.2%) in 2017 in Ethiopia. Of the NCDIs, injuries (17%), cardiovascular diseases (10%), mental illness (9%) and cancer (10%) cause the highest loss of DALYs.

A comprehensive review of the literature on NCDIs in Ethiopia from three demographic surveillance sites, representing both rural and urban communities, and mortality surveillance data from Addis Ababa conducted between 2006 and 2018 showed results comparable to the 2017 GBD estimates, with a higher NCD burden in urban areas than in rural areas (NCDs contributed to 52% and 34% of the disease burden in urban and rural areas, respectively) (18–23).

Our review of studies of common NCDs and their risk factors in Ethiopia revealed a large and diverse burden that varies according to socioeconomic factors (Tables 1 and 2). Tobacco, alcohol and khat use are on the rise in Ethiopia. Even though there is a lack of consensus regarding the evidence associating khat use with NCDs, there is ample data suggesting that its consumption is often accompanied by alcohol and tobacco use, as well as risky behaviours such as unsafe sex and reckless driving leading to road traffic injuries (RTIs) (24–29). Obesity, insufficient physical activity and raised total cholesterol, whilst present in all socioeconomic strata, are most prominent in wealthier groups. Overweight and obesity rates in the wealthiest quintile were 6.6 times higher than the average rates for the other four quintiles (30). Even though the overall rate of overweight/obesity in Ethiopia in 2015 was low (6.3%), even when compared with neighbouring African countries, it is important to highlight the increasing trend, especially among women in general and young children in urban areas (30–32). Rates of tobacco and khat use, heavy alcohol drinking and exposure to indoor air pollution were more common among poorer households (30,33). Exposure to indoor air pollution in Ethiopia is very high, increasing the risk of acute and chronic respiratory conditions, particularly in women, girls and children (34–36). A review of six studies conducted in urban and rural communities in Ethiopia showed variations in the prevalence of household biomass fuel use, ranging from 60% in Addis Ababa (urban) to 100% in rural communities (33,34,36–39). In contrast, fruit and vegetable consumption was reported as being low in Ethiopia, with >98% of individuals displaying inadequate consumption (40). Salt intake in Ethiopia is high (96% of the study participants consume ≥5 g/day), with a mean daily consumption of 8.3 g, which is higher than the WHO recommendation (less than 5 g of salt per day) (41). According to GBD 2016, dietary risks were the leading risk factors for both sexes, accounting for nearly 12% of the total DALYs lost (1,42).

**Table 1:** Prevalence of risk factors for NCDs in Ethiopia by gender, area of residence and wealth status

Risk factors	Study area/type	Male	Female	Urban	Rural	Total	Distribution by wealth quintile (Q)
**Prevalence of** **tobacco use**^	Institution-based (age: 15–30)*	—	—	—	—	8.1%	Q_1_=6.6% Q_2_=4.4% Q_3_=5.1% Q_4_=1.8% Q_5_=3.2%
	Community-based (age: 15–64)*	13.8%	0.3%	—	—	5.8%	
	STEPs survey (age: 15–69)	7.3%	0.4%			4.2%	
**Prevalence of** **alcohol** **consumption**^	Institution-based (age: 14–30)*	—	—	—	—	24%	Q_1_=34.6% Q_2_=37.1% Q_3_=38.9% Q_4_=40.7% Q_5_=48.9%
	Community-based (age: 15–64)*	—	—	—	—	25%	
	STEPs survey (age: 15–69)	46.6%	33.5%			40.7%	
**Prevalence of** **insufficient physical** **activity**∼	STEPs survey (age: 15–69)	8.6%	19.4%	—	—	13.6%	Q_1_=16% Q_1_=14% Q_3_=13% Q_4_=15% Q_5_=35%
**Prevalence of** **overweight or** **obesity**^	Institution-based (age: 5–19)*	—	—	—	—	8.5%	Q_1_=2.3% Q_2_=2.4% Q_3_=2.4% Q_4_=3.3% Q_5_=17.1%
	Community-based (age: 25–64)*	—	—	—	—	13.7%	
	EDHS, 2005 (age: 15–49, women)	—	3.7%	—	—	—	
	EDHS, 2011 (age: 15–49, women and 15–59, men)	2.2%	4.7%			3.4%	
	EDHS, 2016 (age: 15–49, women and 15–59, men)	3.5%	7.6%	17.8%	2.3%	5.7%	
	STEPs survey (age: 15–69)	4.4%	8.8%	12.7%	3.4%	6.3%	
**Prevalence of raised** **blood cholesterol**∼	STEPs survey (age: 15–69)	3.9%	6.8%	7.1%	4.8%	5.2%	Q_1_=4.2% Q_2_=3.3% Q_3_=6.1% Q_4_=6.7%
**Prevalence of khat** **consumption**^	Institution-based*	—	—	—	—	18%	Q_1_=17.7% Q_2_=19.1% Q_3_=16.5% Q_4_=11.9% Q_5_=12.7%
	Community-based*	—	—	—	—	21%	
	STEPs survey (age: 15–69)	21.1%	9.4%			15.8%	
**Prevalence of** **household biomass** **fuel use**	Ethiopia Welfare Monitoring Survey, 2011			87.4%	99.6%	95%	Q_1_=99.3% Q_2_=99.2% Q_3_=94.2% Q_4_=87.7% Q_5_=75.9%
	Community-based*			80%	99.7 %	91%	

Explanations:

* The rates are weighted averages.

^ The disaggregation by wealth quintiles is based on the average from the 2016 STEPs survey and distribution from EDHS, 2016.

Wealth quintile (Q) = Q1 is poorest; Q2 second poorest; Q3 middle; Q4 second richest; Q5 richest.

∼ Disaggregation by wealth quartiles is from the STEPs survey

**Table 2:** Prevalence of NCDs in Ethiopia

NCDs	*Study area/type*	*Urban*			*Rural*			*Total*	*Wealth* quartile*

		*Male*	*Female*	*Total*	*Male*	*Female*	*Total*		
**Raised blood** **pressure**	Institution-based (age: 18–64)*	23.9%	14.7%	21%	—	—	—	21%	Q_1_=18.1% Q_2_=14.6% Q_3_=15.0% Q_4_=17.6%
	Community-based, only urban (age: >15)*	27.9%	25.2%	26.5%	—	—	—	26.5%	
	Community-based, both urban and rural (age: >15)*	22%	21	21.3%	15%	11%	13%	15.8%	
	STEPs survey (age: 15–69)			19.7%			14.9%	15.8%	
**Rheumatic** **heart disease**	School-based: (age: 4–24)*							17‰	
	Community-based: (age: 6–25)*							37.5‰	
**Raised blood** **glucose**	Hospital-based (age: 15–89)	—	—	—	—	—	—	7.8%	Q_1_=2.9% Q_2_=2.2% Q_3_=2.6% Q_4_=3.3%
	Community-based, both urban and rural (age: >35)*	4.3%	5.6%	5.1%	1.7%	2.6%	2.1%	3.5%	
	Institution-based (age: >18)*	5.9%	6.1%	6%				6%	
	STEPs survey (age: 15–69)			3.2%			3.2%	3.2%	

* Disaggregation by wealth quartiles is based on the 2016 STEPs survey results. For rheumatic heart disease disaggregated data by wealth is not available.

The burden of common NCDIs such as hypertension (with prevalence in the adult population ranging from 16% to 27%, and higher rates in urban dwellers than in their rural counterparts) and diabetes (3.2%–8% of the adult population) was high, as was the burden of less common but more severe NCDIs such as rheumatic heart disease (prevalence ranging from 17‰ to 37.5‰ of the population aged 4–24) and cancers in women (40,43–63). Hospital-based studies from the period 1985–2000 documented a rise in the rates of stroke and myocardial infarction in Ethiopia during this period (64,65). More than 40% of strokes in Ethiopia were haemorrhagic, in contrast to the situation in western countries (<15%) (66,67).

Cancer, especially breast and cervical cancer, is a staggering public-health problem in Ethiopia. There were an estimated 65,000 cancer cases in Ethiopia in 2015, based on projections from the population-based cancer registry in Addis Ababa, and it was estimated that two-thirds of the cancers occurred in women (63). These figures could be an underestimate, as population-based cancer registries are highly dependent on access to health-care services. The ten commonly occurring cancers in Ethiopia were found to be cancers of the breast and cervix, colorectal cancer, non-Hodgkin lymphoma, leukaemia and cancers of the prostate, thyroid, lungs, stomach and liver.

Mental, neurological and substance-use disorders in Ethiopia account for 25.6% of all years lived with disability (YLD) in 2017, affecting nearly 30% of the Ethiopian population at any given time (1). Common mental disorders were the most prevalent mental-health problems (21.6%), followed by less common but more severe conditions such as major depression (6.8%), schizophrenia (0.5%), bipolar disorder (0.5%) and epilepsy (0.52%) (68–71). In an Ethiopian rural community, schizophrenia and depression alone accounted for over 11% of the burden of diseases (72).

Furthermore, injuries — particularly road traffic accidents, falls and interpersonal violence - accounted for 8% of total DALYs lost in Ethiopia in 2017 (1). Chronic respiratory diseases only constituted 1.6% of the total DALYs lost in 2017 in Ethiopia (1). Regardless of this, several local studies showed that asthma was a fairly common health problem in Ethiopia, being prevalent in 1.5–3% of the population (73–75). The prevalence of COPD is unknown in Ethiopia, even though hospital-based studies indicate that the problem is fairly common (76,77). Eye-health problems are major causes of disability in Ethiopia. Cataract is responsible for nearly 640 thousand cases of blindness, and for an additional 1.25 million cases of poor vision in the country (78). Digestive diseases, surgical conditions and musculoskeletal disorders also play a large role in morbidity and mortality in relation to NCDIs. Surgical conditions were among the most common causes of admissions to secondary and tertiary hospitals. According to global estimates, East Africa is one of the countries with the greatest need for surgical procedures, with a reported 6,145 procedures per 100,000 people (79). Most surgery is related to injuries, malignancies and acute abdominal emergencies in adults and congenital abnormalities and acute abdominal emergencies in children (80).

**Economic impact of NCDIs**: For individuals and their households, NCDs can have devastating long-term economic consequences. A systematic review of the economic effect of NCDs on households in LMICs showed that NCDs placed a substantial economic burden on patients and their families in all socioeconomic strata, particularly in the poorest sector of the population. Direct medical expenses for drugs, diagnostics, outpatient visits and hospitalisation were the major components of out-of-pocket (OOP) costs (4). The cost of transportation to access care was also substantial. The highest incidences of catastrophic health expenditure were reported for low-income patients with CVD (up to 92%), followed by cancer (68%) and epilepsy (64%). Such high OOP costs could cause poverty in a substantial proportion of households (4). A lack of health insurance was the main predictor of economic burden for NCDs, though even people with health insurance incur catastrophic health expenditure when they have low incomes and high co-payments or limited coverage (4).

There is a shortage of resources for health care in Ethiopia, with a per capita expenditure of US$33 in 2016/2017 (7). Despite NCDs comprising nearly 40% of the total disease burden (which is expected to increase even more over the next couple of decades), only 11% of total health spending in 2016/17 went on NCDs. Most of the spending (68%) on NCDs in Ethiopia was financed by OOP expenditures from households (7). Government was responsible for only 30% of NCD expenditure, whilst contribution by donors for such services was negligible, at only 1%. Health-insurance coverage in Ethiopia is very low (<10% of the population are covered by community-based health insurance) (81). Reliance on OOP payments at the point of care is an obstacle to access to care, particularly for poor households and those who opt for treatment. They may be confronted with the tragic consequence of sending their families into poverty. Most patients with NCDs in Ethiopia do not access care, and those who do are highly likely to suffer financial hardship (40,82). Of the patients with cardiovascular diseases in Addis Ababa who sought care in health facilities, 27% had experienced catastrophic health expenditure (CHE), and the prevalence of CHE was even higher in low-income households of patients residing outside Addis Ababa (82). In addition to economic consequences of OOP expenditure, the economic burden of NCDIs on households includes loss of employment for patients and care providers and the increased cost of living with disability (4).

**Priority setting for essential interventions for NCDIs**: A task faced by many LMICs is identification of high-priority interventions for NCDIs and fair prioritisation of introduction or scale-up of these interventions. The gap between health requirements for NCDIs and economically feasible provision is a substantial challenge for LMICs, and likewise for Ethiopia. Health care for chronic diseases has largely been neglected, in comparison with interventions that prevent, diagnose and treat malnutrition, infection and maternal and childhood diseases.

After establishing the major NCDI conditions in Ethiopia, the Commission identified an initial list of 235 potential health-sector interventions for implementation or scaleup in the Ethiopia health system. The commission used the WHO Consultative group on Equity and Universal Health recommendations to prioritize NCDI interventions: cost-effectiveness, fair distribution (priority to the worse-off) and FRP (14). Evidence of cost-effectiveness was available for 80 of these interventions (17,83). Screening for cervical cancer and treatment of pharyngitis in children to prevent rheumatic heart disease are examples of very cost-effective interventions with cost-effectiveness ratios below US$100 per DALY averted. In contrast, acute management of stroke and peritoneal dialysis was the least cost-effective of the interventions considered, with cost-effectiveness ratios above US$30,000 per DALY averted. A total of 90 interventions (including essential surgery) were recommended as top-priority NCDI interventions that should be scaled up or implemented during 2019–2023 (the list of top priority health sector NCDI interventions and their delivery platforms is included in table 3). Around 70 interventions were classified as medium-priority interventions that should be scaled up to 30% effective coverage over the next five years and to 80% by 2030, and 60 were classified as low-priority interventions. The remaining interventions were not considered relevant in the Ethiopian context or were redundant with other interventions. The additional annual cost for scaling up these interventions was estimated to be US$549m, corresponding to US$4.70 per capita (Table 4).

**Table 4:** Incremental costs by major category, 2019–2023. Costs are reported in US$1,000

*Interventions*	*Implementation year (all amounts in US$)*				

	*2019*	*2020*	*2021*	*2022*	*2023*
Cancer	$7,494	$15,406	$23,748	$32,496	$41,649
Cardiovascular diseases and diabetes	$46,438	$95,429	$147,017	$201,292	$258,307
Mental, neurological and substance-use disorders	$12,231	$25,773	$40,708	$57,106	$75,025
Surgery	$22,377	$45,865	$70,526	$96,424	$123,622
Other interventions: provision of glasses for severe refractive disorders	$127	$258	$392	$529	$668
Total intervention cost	$88,667	$182,731	$282,392	$387,846	$499,271
Programme cost	$8,867	$18,273	$28,239	$38,785	$49,927
TOTAL COSTS	$97,533	$201,004	$310,631	$426,631	$549,198
Cost per capita (US$ per capita)	$0.9	$1.8	$2.8	$3.7	$4.7

Additionally, multi-sectoral interventions designed to reduce population-level behavioural and environmental risk factors (e.g. tobacco and alcohol use, air pollution and excessive sugar consumption) are available in the commission's report (13). Many of these are policy interventions that fall into four broad categories, namely taxes and subsidies, regulations and related enforcement mechanisms, the built environment and informational interventions. Some of these intersectoral interventions could be cost-saving, and others could potentially generate more resources for health. We thus did not estimate the cost of implementing these interventions.

**Estimated health impact of NCDI interventions**: The Commission made a conservative estimate that, if fully implemented, the Highest Priority Package for NCDI services (excluding multi-sectoral interventions) could raise life expectancy at birth from 64.4 to 65.2 (i.e. by 0.8 years), and avert approximately 41,000 premature deaths by 2030 (16,84). These modest results should be seen as part of the bigger picture. Larger investments on NCDIs are needed and long-term strategies by means of which full implementation of all essential UHC packages could provide substantial health gains and contribute to attainment of the SDG health targets.

**Fiscal space, resource generation and mobilisation, and financing**: As described above, there is a shortage of health-care resources in Ethiopia. The Ethiopian government recognises the obligation to optimize domestic resources for health in order to achieve the progressive realisation of UHC (85). The Commission thus carried out a simple fiscal-space analysis. Fiscal space can be understood as the ‘budgetary room’ that can allow government to devote resources to specific services or activities without endangering the sustainability of its financial position (86). The budgetary room is largely determined by three factors: economic growth, the level of total government expenditure and the percentage of total government expenditure devoted to health.

**Figure 1a-c:**
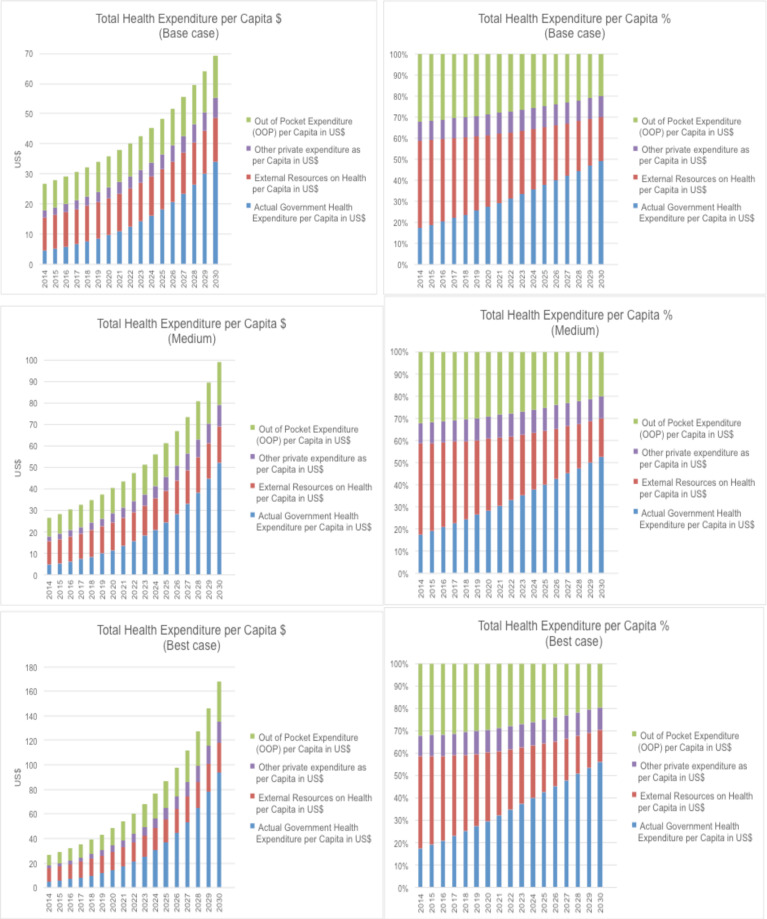
Total health expenditure per capita projections, 2014 – 2030. (left: in US$; right: in percent)

With conservative assumptions and targets of GDP growth of 3.5%, total government health expenditures of 4% of the GDP and NCDI expenditure 20% of the total health expenditure (base case), the Commission estimated that by the end of the first five-year period (2023), there will be US$2.40 extra per capita for NCDI services from the government budget. However, in a best-case scenario of GDP growth of 7%, total government health expenditures of 6% of the GDP and NCDI expenditure 30% of the total health expenditure, there would be an extra US$4.50 for the toppriority package of NCDI services. The potential fiscal space per capita in this best-case scenario approximates the estimated per capita cost of US$4.70 (needed in the year 2023) for the recommended top-priority NCDI services. The three variants for possible targets and budget expansion paths for total (THE) and government health expenditures (GHE) that would be compatible with a commitment to move towards UHC are illustrated in Figure 1a-c.

**Recommendations from the Ethiopia NCDI commission**: Ethiopia is facing a substantial burden from NCDIs such as ischaemic heart disease, stroke, diabetes, major depressive disorders, cancer and chronic respiratory diseases, the incidence of which is increasing at an alarming rate (1,5). Disaggregation of the burden of NCDI risk factors by socioeconomic strata demonstrated a mixed pattern. Obesity, a low level of physical activity and raised total cholesterol are more prominent in wealthier quintiles, whilst tobacco, khat use, heavy alcohol consumption, and exposure to indoor air pollution are more common in poorer quintiles. The distribution of NCDIs is likely to follow the pattern of associated risk factors. The NCDI burden is rapidly expanding as a result of a severely under-resourced (in terms of both trained health-professional manpower and finance) and inequitable health system already overwhelmed by communicable, maternal and nutritional disorders. Dealing with the threat posed by NCDIs and injuries requires a focused, accelerated expansion of health systems delivering maximum health, fairly distributed, with financial risk protection (87). The strategic options include expansion of access to treatment as well as promotion of health and disease prevention. There are proven cost-effective interventions that are well suited to implementation in low-income settings such as Ethiopia (88). The Commission has identified 90 cost-effective interventions for implementation in Ethiopia, including 23 multisectoral interventions. Most of the preventive and curative interventions can be integrated with existing services using primary health care as the main delivery platform. Beyond increasing access to care, this approach has the additional advantage of ensuring equitable access to care, especially in the rural population, where most poor people in Ethiopia live.

The primary health-care (PHC) system in Ethiopia is mainly organised around acute care delivery. In recent years the concept of chronic care has been introduced in PHC facilities through HIV service delivery, from which several implementation lessons could be learned for the delivery of NCDI services. These lessons include implementing decentralised care in the PHC, a multidisciplinary approach to care through task-shifting and task-sharing, simplification of protocols and guidelines, use of standardised essential drugs and diagnostic packages, laboratory networking, harmonised recording and reporting systems and facilitated referral mechanisms. The main strategies for prevention, treatment and care of HIV/AIDS are early detection and monitoring of risk factors, population-based interventions, a care continuum, regular monitoring of treatment adherence and psychosocial interventions. These are well-established strategies for chronic care, which could inform NCDI services. Investment in a health-system approach to NCDIs should be the way forward, as fragmented, disease-based approaches will not enable the country to respond to these emerging health problems. Substantial constraints are anticipated across each of the six key health-system components during NCDI services integration and scale-up in Ethiopia. Governance and technical support for NCDIs should be devolved and capacitated at the regional level in order to ensure appropriate decision-making and implementation of NCDI initiatives. Furthermore, the wide-ranging risk factors and the impact of NCDIs on Ethiopian society, as well as the fact that prevention and control of NCDIs require the involvement and commitment of the government and all stakeholders, including the community, call for the establishment of a national multisectoral committee on NCDIs chaired by the Prime Minister or Deputy Prime Minister, in order to guide and organise NCDI efforts. The Ministry of Health has a critical leadership role in spearheading the government's commitment. A summary of the Commission's recommendations is presented in Box 1.

**Box 1: Key recommendations by the National NCDI commission.**

1. ***Policy, planning and oversight*** Establish a national multisectoral committee on NCDIs, chaired by the Prime Minister or Deputy Prime Minister, to guide and organise NCDI efforts
  - Strengthen the NCD Prevention and Control Unit at federal level
  - Establish NCD Prevention and Control Units at regional level
  - Protect the implementation of public-health policies for NCDI prevention and control against interference by vested interests through comprehensive legislation and enforcement of national laws and regulations.
2. ***Finance***
  - Allocate an increased percentage of gains from economic growth to health; rapidly move government spending on health towards 5% of GDP
  - Mobilise additional resources, including improved efficiency, improved taxation and sin taxes (tobacco, alcohol), and specifically consider tax on sugar-sweetened beverages
  - Move towards allocating 30% of total government spending (GHE) to NCDIs by 2030
  - Plan for reduced external funding (at least in relative terms), as the country develops economically and transitions to the status of lower-middle-income country
  - Reduce out-of-pocket to a maximum of 20% of total health expenditure
  - Harmonise community-based health insurance, and if possible social health insurance, with public health-sector priorities
  - Development of partners for engagement in generating evidence and supporting implementation of costeffective and equitable interventions for NCDs and injuries, based on national needs, in order to maximise the effectiveness of aid
3. ***Service integration***
  - Strengthen the health system at all levels, emphasising primary care, and finance the national set of NCDI services, interventions and health promotion, focusing on prevention and early detection, as well as curative, rehabilitative and palliative care
  - Low-resource countries such as Ethiopia can prevent and manage NCDs and injuries by integrating a high-priority package of interventions into existing platforms; the package's interventions should be cost-effective, target the worse-off and provide financial risk protection
  - Implement scale-up of health personnel as planned in HSTP, with a special emphasis on the resources needed for NCDI services
  - Improve the training of health workforces and the scientific basis for decision-making through NCD-related research and partnerships
  - Develop, train and implement the top eleven clinical guidelines for NCD and injury services (treatment of childhood cancer, early treatment of breast cancer, basic palliative care, treatment of acute pharyngitis in children to prevent rheumatic fever, psychosis {basic psychosocial support and anti-psychotic medication}, epilepsy {follow-up and anti-epileptic medication}, treatment for substance use disorder {alcohol and tobacco}, primary prevention {statins and antihypertensive} for those with an absolute 10-year risk of CVD>10%, management of acute heart failure with diuretics and non-invasive positive-pressure ventilation, and detection and treatment of asthma)
4. ***Strategic information, target-setting, monitoring and evaluation***
  - Strengthen integrated national surveillance systems for NCDIs, including vital registration systems capable of reporting cause of death, cancer registers and risk-factor monitoring
  - Refine existing NCDI indicators and expand to include other priority NCDIs in future revisions of HIS in order to monitor the epidemiology and service coverage of NCDIs
  - Track results of NCDI interventions by monitoring and reporting on the attainment of the 9 national targets (based on the 9 global targets — details are to be found in Section 5 of this report).
  - Mobilise and track domestic and external resources for NCDI prevention and control. Improve NCDI subaccounts in next round of National Health Accounts report.
5. ***Education and advocacy:*** Achieving global as well as local commitment and action against NCDIs requires sustained advocacy. Alongside actions directed at changing social, environmental and economic conditions that impact health, mobilising the public and educating them on a healthy lifestyle will substantially contribute to the prevention of NCDIs. Advocate for improved NCDI resources and services.

There is shortage of well-trained health professionals in Ethiopia, where there are only 0.96 per 1000 population, which is far less than the 4.45‰ recommended in order to meet the SDG health targets. Besides the shortage of human resources, inadequate knowledge and skill in NCDI case management are among the expected challenges for the health workforce during decentralisation of NCDI services. The shortage will be more pronounced in terms of staff attrition, with trained staff with their NCDI skills leaving institutions, leading to compromised case management and inconsistent completion of client-NCD risk-assessment forms, which may exacerbate the long-term monitoring of NCD risk factors. Even though the general provision of health-care professionals relative to the population is low, recent studies suggests that there is much to be gained by improving the efficiency of health facilities in Ethiopia (89,90). A study of health-centre efficiency showed a low average number of outpatient equivalent visits per medical staff member per day (3.7) compared with the figures for Kenya (7 outpatient equivalent visits per medical staff member per day) and Ghana (4 outpatient equivalent visits per medical staff member per day) in 2011. Furthermore, health centres in Ethiopia were generously staffed, with a high average number of medical staff members (26), compared with the minimum requirement of 12, pointing to a potential overstaffing problem relative to patient volume. The generous staffing and the low efficiency level could potentially be utilised for NCDI service delivery in Ethiopia, with additional efforts to address challenges related to quality improvement. Considering these facts and other challenges related to the health workforce, the NCDI commission recommends provision of targeted training in NCDI prevention, diagnosis and management for the available staff, aiming at continuous professional development, as well as development and utilisation of user-friendly clinical guidelines, treatment protocols and standard operating procedures. The long-term solution should include scaling up transformative high-quality education and lifelong learning, so that all health workers will have the skills to match the population's health needs and will be able to work to their full potential.

Generation of sufficient resources is critical to the delivery of health-care services. The fiscal-space analysis conducted by the Commission demonstrates that with more commitment from the government, attainment of 30% coverage of high-priority interventions is possible in the best-case scenario. In addition to economic growth, there are other ways of increasing fiscal space for government health expenditure to 5–6% of GDP. They include increased mobilisation of domestic resources, intersectoral reallocation and efficiency gains. As for increased mobilisation of domestic resources, particularly important options to be considered are improved taxation and other forms of revenue collection, including increased taxation of tobacco and alcohol (91). Such an increase would be likely not only to increase revenue, although only to a certain degree for tobacco, since consumption is low, but also to improve future health within the population. With respect to intersectoral reallocation, a related strategy is reduction or elimination of energy subsidies and other unwarranted subsidies. This may increase the fiscal space for public spending on high-priority health services. Other types of innovative financing should also be explored. As for efficiency gains, there are many promising strategies to be pursued. The 2010 World Health Report lists ten leading causes of inefficiencies that could be addressed: underuse of generic drugs; use of substandard and counterfeit medicines; inappropriate and ineffective use of medicines; overuse or supply of equipment; inappropriate or costly staff mix and unmotivated workers; inappropriate hospital admissions and length of stay; inappropriate hospital size (low use of infrastructure); medical errors and suboptimal quality of care; waste, corruption, and fraud; and inefficient mix or inappropriate level of strategies (92). Finally, the commencement of community-based health insurance in Ethiopia and the government's endeavour to launch social health insurance could serve as an important platform for strengthening the health system and improving delivery of quality care for NCDIs (85,93). Expansion of such a platform could not only provide the necessary financial protection for the population and shield against health-care-related impoverishment, but it could also create additional fiscal space with which to expand the essential package of services.

The NCDI burden in Ethiopia is increasing and is expected to grow at an alarming rate in the next couple of decades. A targeted investment in cost-effective interventions could result in substantial reduction in premature mortality and may be within the projected fiscal space of Ethiopia. Innovative financing mechanisms, multi-sectoral governance, regional implementation, and an integrated service delivery approach mainly using primary health care are required to achieve these goals.

## Abbreviations

AIDS: Acquired Immuno-deficiency Syndrome; CHE: Catastrophic Health Expenditure; COPD: Chronic Obstructive Pulmonary Disease; CVD: Cardio-vascular Disease; DALYs: Disability-adjusted Life Years; GBD: Global Burden of Diseases; GDP: Gross Domestic Product; GHE: Government Health Expenditure; GNI: Gross National Income; HIV: Human Immunodeficiency Syndrome; LMICs: Low-and Middle-Income Countries; NCDs: Noncommunicable Diseases; NCDIs: Noncommunicable Diseases and Injuries; OOP: Out-of-pocket; PHC: Primary Health Care; RTIs: Road Traffic Injuries; SDGs: Sustainable Development Goals; THE: Total Health Expenditure; UHC: Universal Health Coverage; UN: United Nations; US: United States; YLD: Years Lived with Disability.
